# Diminished neural network dynamics after moderate and severe traumatic brain injury

**DOI:** 10.1371/journal.pone.0197419

**Published:** 2018-06-08

**Authors:** Nicholas Gilbert, Rachel A. Bernier, Vincent D. Calhoun, Einat Brenner, Emily Grossner, Sarah M. Rajtmajer, Frank G. Hillary

**Affiliations:** 1 Department of Psychology, The Pennsylvania State University, University Park, PA, United States of America; 2 Social and Life and Engineering Sciences Imaging Center, University Park, PA, United States of America; 3 The Mind Research Network, Albuquerque, NM, United States of America; 4 Department of Electrical and Computer Engineering, The University of New Mexico, Albuquerque, NM, United States of America; 5 College of Information Science and Technology, The Pennsylvania State University, University Park, PA, United States of America; 6 Department of Neurology, Hershey Medical Center, Hershey, PA, United States of America; University of Bristol, UNITED KINGDOM

## Abstract

Over the past decade there has been increasing enthusiasm in the cognitive neurosciences around using network science to understand the system-level changes associated with brain disorders. A growing literature has used whole-brain fMRI analysis to examine changes in the brain’s subnetworks following traumatic brain injury (TBI). Much of network modeling in this literature has focused on static network mapping, which provides a window into gross inter-nodal relationships, but is insensitive to more subtle fluctuations in network dynamics, which may be an important predictor of neural network plasticity. In this study, we examine the dynamic connectivity with focus on state-level connectivity (state) and evaluate the reliability of dynamic network states over the course of two runs of intermittent task and resting data. The goal was to examine the dynamic properties of neural networks engaged periodically with task stimulation in order to determine: 1) the reliability of inter-nodal and network-level characteristics over time and 2) the transitions between distinct network states after traumatic brain injury. To do so, we enrolled 23 individuals with moderate and severe TBI at least 1-year post injury and 19 age- and education-matched healthy adults using functional MRI methods, dynamic connectivity modeling, and graph theory. The results reveal several distinct network “states” that were reliably evident when comparing runs; the overall frequency of dynamic network states are highly reproducible (r-values>0.8) for both samples. Analysis of movement between states resulted in fewer state transitions in the TBI sample and, in a few cases, brain injury resulted in the appearance of states not exhibited by the healthy control (HC) sample. Overall, the findings presented here demonstrate the reliability of observable dynamic mental states during periods of on-task performance and support emerging evidence that brain injury may result in diminished network dynamics.

## Background

One approach to understand the consequences of traumatic brain injury (TBI) on brain functioning has been the use of functional MRI (fMRI) methods, with now more than 25 years since the first fMRI studies [1,2]. Since these early seminal studies, the change in the landscape of this literature is reflected in the development of novel methods and much recent work has turned the focus toward understanding interactive nodes of large-scale neural networks. The first application in TBI examined network plasticity in global network metrics such as the local communication, or clustering, and distance between nodes throughout the network, or path length [3]. More recent work focused on the role of critical subnetworks and the most highly connected nodes, or hubs, in the expression of plasticity post injury. For example, several studies have revealed altered responses in parts of the default mode network, a network associated with regulation of internal states and memorial functioning [4,5] and others have shown increased responses in the executive control networks [6–10]. Furthermore, investigators have observed changes within the salience network (SN), which is a network that commonly operates between these systems as a liaison between states of self-reflection (default mode network; DMN) and goal-directed behavior (executive control network; ECN) [11]. Enhanced connectivity in the SN has been demonstrated over the course of recovery from injury [8], and connectivity between the anterior insula and ACC within the SN have been linked to attentional capture and cognitive outcome in TBI [12,13].

Findings in the TBI literature using connectivity modeling have not always converged, with results revealing enhanced involvement of large subnetworks, loss of connectivity in larger subnetworks, or some combination of the two after injury [14,15]. A call for study reproducibility has come to the forefront in critiques of brain imaging research over the past several years, with increasing awareness that much of what is observed using fMRI methods can be altered by a number of investigator-dependent factors such as brain parcellation, thresholding, and edge definition [16–18]. Attempts to verify the reliability of results using machine learning or other approaches have been met with some success [19], but much of the research to date has used data collection runs that are relatively short, reducing test-retest reliability [20].

Moreover, the definition of connections has been almost universally static, which undermines the dynamic nature of the signaling between the brain’s large subnetworks [14,21]. Static connectivity quantifies the relationship between the signals emanating from two spatially discrete regions as a single value to indicate the covariance (e.g., correlation). Dynamic network approaches [22] attempt to model the connectivity between regions over smaller windows of time in order to determine intermediary brain states that neural systems move through during periods of task and rest (recently applied in mild TBI [22].

### Study goals and hypotheses

Our objective is to understand the influence TBI has on large-scale neural networks with focus on distinct brain states assessed using dynamic functional connectivity (dFC) approach. In doing so, we have three primary goals in this study. First, given the novelty using dFC methods in moderate and severe TBI and the goal to demonstrate reproducibility, we compare the observed brain states across two long data collection runs (2 x ~8 minutes) to serve as a test-retest reliability of dynamic state data. Second, we examined traditional network “hubs” and sites where TBI has resulted in enhanced involvement to determine if there are localized “drivers” of network states and transitions [23]. Prior work has shown clear evidence for disturbance in large-scale networks, with findings largely focused on network hubs in parts of the DMN, ECN, and SN [14,24]. Elegant work using functional and structural imaging methods has isolated parts of the SN, including anterior insula, as a critical marker for information processing deficits evident in TBI [13]. We have made similar observations [25] so there is reason to focus on these three networks during dynamic connectivity modeling to determine their role in the efficiency of information processing across brain states. Therefore, we aim to isolate the most highly connected nodes in each of the network states and determine if these state-dependent hubs drive networks toward distinct states and if they predict cognition in TBI. Finally, we aim to determine if TBI results in distinct connectivity states and quantify the number of transitions between states over the course of data acquisition as an indicator of network dynamics.

Given these goals, the primary hypotheses are threefold. *Hypothesis 1*: We predict that dynamic states are relatively stable resulting in high reliability in both samples when comparing the frequency of states over the two runs of data collection. *Hypothesis 2*: Based upon the TBI literature demonstrating an important role of connectivity changes in the medial frontal regions in the DMN [4,5,26], prefrontal cortex in the ECN [4], and the insula within the SN [4,12,13,25] we expect that these critical hubs will act as “drivers” for the states differentiating the TBI from a matched-control sample. *Hypothesis 3a*: TBI results in elevated behavioral variability and we anticipate that dFC will predict these behavioral findings showing that brain network states observed in TBI-affected individuals will have greater network dynamics (operationalized as the number of transitions between states). *Hypothesis 3b*: As a corollary to Hypothesis 3a, we anticipate that as a natural consequence of increased network connectivity, network dynamics (i.e., state transitions) will decrease due to diminished opportunities for network variation.

## Materials and methods

Subjects included 23 individuals who sustained moderate to severe TBI and 19 healthy controls of comparable age and education (see Table 1 for demographic information). All data collection, including MRI data acquisition and cognitive testing, occurred on the same day for each subject. Moderate to severe TBI was defined as a Glasgow Coma Scale (GCS) at time of injury between 3–8 (severe injury) or 9–12 (moderate injury) [27]. Subjects were excluded if they were receiving treatment for concomitant injuries (e.g., orthopedic injuries or injury to the spinal cord) that would make it difficult for them to remain comfortable and still in the MRI environment. When available, the outcome of acute CT/MRI findings are reported in Table 2.

**Table 1:** Demographic information.

	n	Mean age in years (sd)	Mean education in years (sd)	Gender	GCS (sd)	Mean time post-injury in years (sd)
**TBI**	23	31.74 (12.90) range: 18–69	13.00 (3.08) range: 12–18	12 F, 11 M	7.85 (4.58) Range: 3–15	4.24 (5.84) Range: 0.24–22.8
**HC**	19	34.63 (12.80) range: 18–60	13.68 (1.77) Range: 12–18	9 F, 12 M	NA	NA

**Table 2:** Injury severity, mechanism of injury and brain imaging findings for all TBI subjects.

S#	Glasgow Coma Scale score	Mechanism of Injury	Injury Characteristics
**1**	7	Fall	Edema in frontal pole bilateral, paracingulate gyrus, and bilateral cerebral white matter
**2**	unknown	MVA	No acute findings; A few weeks post injury SPECT and PET scans revealed bilateral frontal and temporal lobe and cerebellar findings
**3**	unknown	unknown	Small to moderate left temporo-parietal subdural hematoma with pneumocephalus. Diffuse left cerebral edema and small subarachnoid blood along the superior interhemispheric falx. Biparietal cephalohematomas.
**4**	3	MVA	MRI scan was interpreted to show a shear injury including the corpus callosum, periventricular regions, and bilateral temporal regions.
**5**	unknown	Ped v. Tractor trailor	Intraventricular hemorrhage in bilateral lateral ventricles (right greater than left), the left temporal horn, the third and the right fourth ventricles. Punctate bifrontal contusions and hyperdensity adjacent to the temporal horn and adjacent to the falx is consistent with contusion. Facial fractures.
**6**	15 (later intubated)	Ped vs. car	Debris in the soft tissues anterior to the skull with large skin lacerations overlying the frontal aspect of the skull. There is no evidence of acute bony fracture.
**7**	3	Fall	Small IPH within the high left frontal lobe. Intraventricular hemorrhage in occipital horns bilaterally, left greater than right. Small subdural along the posterior interhemispheric fissure and tentorium. Facial fractures.
**8**	unknown	MVA	Diffuse bilateral edema in the frontal cerebral cortex, isolated bleeding in the insular cortex, frontal pole and inferior frontal gyrus
**9**	unknown	MVA	Isolated edema in the frontal orbital right cerebral cortex/white matter
**10**	unknown	MVA	Diffuse bilateral bleeding in the frontal lobe, medial bilateral intracalcarine/precuneous cortex bleeding, isolated temporal gyrus bleeding
**11**	unknown	MVA	Edema isolated to right temporal lobe, bilateral bleeding mostly right frontal pole/orbital cortex
**12**	8	Fall	Left frontotemporal epidural hematoma, with adjacent IPH, scattered SAH in the sulci of the inferior frontal lobe and anterior temporal lobes; additional IPH in bilateral inferior frontal lobes and left temporal lobe; left to right midline shift of 6mm;
**13**	9 (3-induced coma)	MVA	Multiple signal drop-out in T2* compatible with hemosiderin and DAI throughout the brain parenchyma bilaterally predominantly centered in the frontal, right parietal and left temporal lobes. There is mild sulcal enlargement advanced for age of the patient.
**14**	14	MVA	bilateral bleeding in the medial dorsal region frontal gyrus, bleeding in left cerebellum, bilateral bleeding in the occipital pole, isolated bilateral bleeding throughout the cortex
**15**	14	Bicycle accident	Subarachnoid hemorrhage within the convexity sulci of the left frontal lobe, parietal lobe, and sylvian fissure. Subarachnoid hemorrhage in right posterior frontal and temporal lobes.
**16**	6	MVA	Surgically removed left temporal epidural hematoma post injury. Cerebral contusion in bilateral frontal lobes, left parietal lobe and left temporal lobe. Nearly complete effacement of the convexity sulci, sylvian fissures resulting in subtle shift to left.
**17**	3	MVA	Small left frontal subarachnoid hemorrhage. Bifrontal subcortical foci of hyperdensity suggesting DAI. Left intraventricular choroid acute hematoma. A small left frontal parasagittal hyperdensity is seen also suggesting of a subdural acute hematoma.
**18**	3	MVA	Small left parietal subdural hemorrhage (2 mm). Small scattered punctate foci of intraparenchymal hypodensities throughout the cerebral hemispheres (e.g., frontal)
**19**	12	Fall	Fronto-temporal intraparenchymal hemorrhages, with subarachnoid hemorrhage. 2 mm midline shift to the right. Midline shift measuring approximately 4 mm.
**20**	15	Fall	Right subdural and subarachnoid hemorrhage; small pectechial hemorrhage within the right lateral frontal region. No midline shift. Left temporal bone fracture.
**21**	3	Fall	Bilateral medial bleeding in the supermarginal gyrus, cingulate gyrus, and insular cortex
**22**	unknown	Ped v. car	Hemorrhage and edema in bilateral frontal pole and left orbital cortex
**23**	unknown	Fall	Isolated areas of edema in right frontal pole and precental gyrus, and in insular cortex

**Note**: Table 2 findings were gathered from medical reports during acute MRI/CT scans when available or by structural scan review by a board certified radiologist during study completion. **Abbreviations**: DAI: diffuse axonal injury, SAH: subarachnoid hemorrhage, IPH: intraparenchymal hemorrhage, mm: millimeters, MVA: motor vehicle accident, Ped: pedestrian, PET: positron emission tomography, SPECT: single photon emission tomography

The research presented in this study was approved by the institutional review board and the Pennsylvania State University Office of Research Protections.

### Behavioral data

All study participants were administered a neuropsychological battery of tests to assess level of cognitive functioning. The battery was designed to examine cognitive domains associated with deficits in TBI, focusing on the domains of attention and working memory [28]. In order for the TBI sample to be capable of reliably completing this battery at least 3-months post injury, the battery was focused on those tests most sensitive to deficits following TBI, including processing speed and working memory. Tests administered included the Visual Search and Attention Test (VSAT), WAIS-III Digit Span (Forward, Digits-F and Backward, Digits-B), Trail Making Test B (TMT), and Stroop Color and Color-Word (Stroop). Table 3 provides mean and standard deviation for cognitive testing for both groups.

**Table 3:** Neuropsychological performance of TBI group. Mean (sd) raw score.

	Stroop C Time	Stroop C Total	Stroop CW Time	Stroop CW Total	Digit Span F	Digit Span B	Trails A	Trails B	VSAT
**TBI**	67.35 (22.35)	110.35 (6.24)	113.79 (12.33)	95.58 (24.31)	10.14 (2.15)	6.81 (2.16)	26.35 (10.52)	66.52 (40.63)	56.90 (13.22)
**HC**	58.25 (11.79)	111.76 (0.97)	108.24 (14.39)	104 (11.61)	11.12 (1.97)	6.84 (2.36)	23.94 (8.33)	57.50 (24.80)	59.74 (10.84)

Abbreviations: Stroop C (color); Stroop CW: color word, Span F: forward; Span B: backward; VSAT: visual search and attention task

#### fMRI task procedures

**Fig 1:**
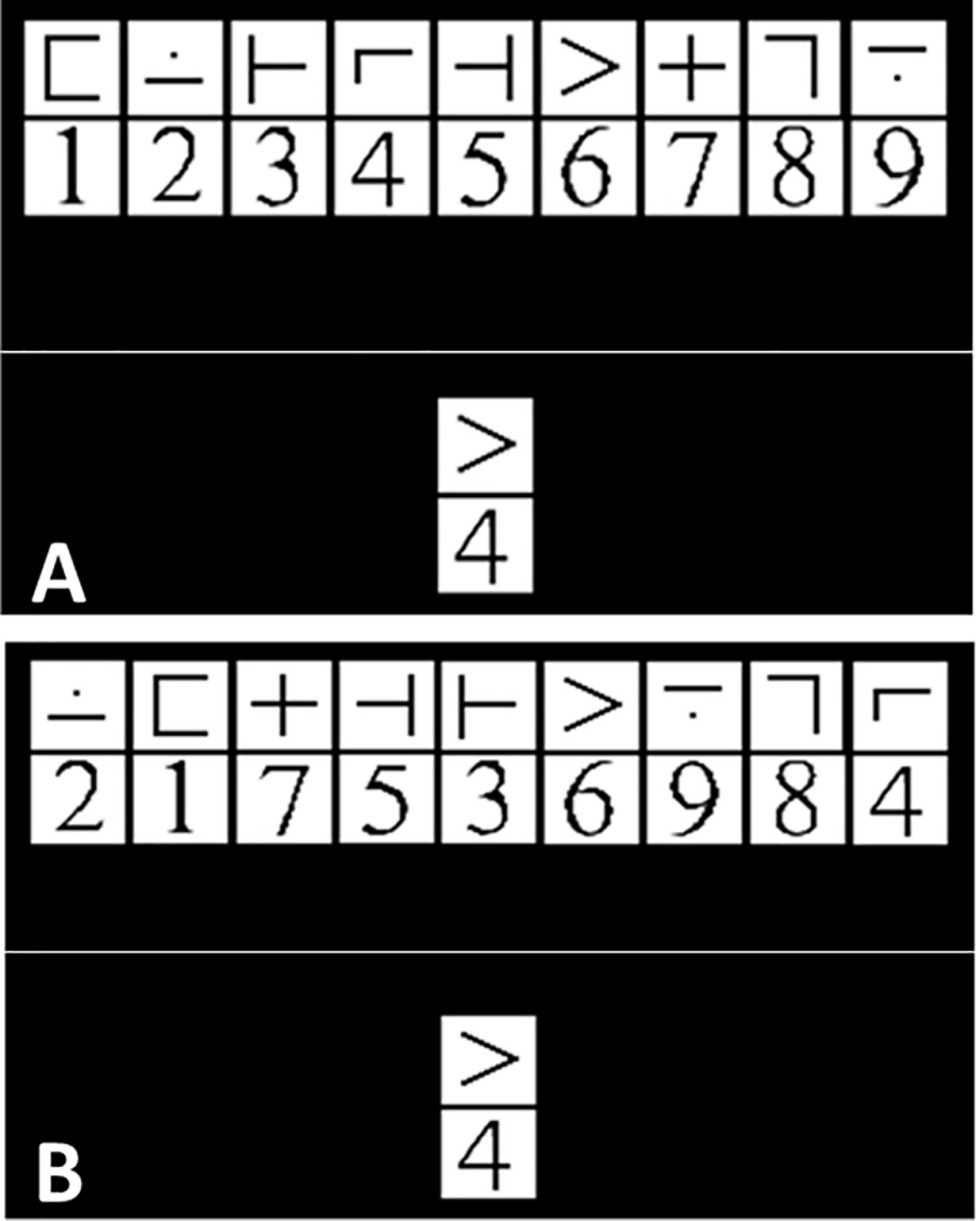
Modified digit symbol modalities task. **A.** Level 1 or 2 of task as it appears in the scanner. Level 1 the symbol-digit pairing remains the same. Level 2, the symbol-digit pairing varies. **B**: Level 3 of the task where symbol-digit pairing in the key code is not in numerical order.

All study participants underwent MRI scanning at Pennsylvania State University, University Park, PA or Hershey Medical Center in Hershey, PA. During MRI data acquisition, subjects completed a cognitive task demanding working memory and rapid information processing task based upon an adaptation of a previously used modified version of the digit symbol substitution test in the MRI environment (mDSST; [29]). See Fig 1 for schematic representation of the task. While only two runs were analyzed for the purposes of this study, participants performed four 7 minute 52 second runs in an event-related design including 244 volumes and 59 “on-task” trials per run and ~20 on-task trials per load level. Participants were equipped with a response trigger for each hand (Nordic Response Grip triggers; NordicNeuroLab). Stimulus presentation and response period occurred within a jittered event-related design and lasted for a total of 3.5 seconds, during which time participants viewed an array of 9 symbol and digit (1 through 9) pairings as well as one “target” symbol digit pairing below it. They were instructed to respond with the right trigger if the bottom stimulus pairing was present in the array above and with the left trigger if the bottom stimulus pairing was absent. To achieve differences in performance and increase challenge, there were three levels of difficulty within each run with each difficulty level taking a total of 2.33 minutes, including time between trials, per run. The first level involved constant symbol-digit target pairings with a constant symbol array to maximize learning. The second level involved random symbol-digit target pairings with a constant symbol array. The third level involved symbol digit pairings in random numerical order and with a random symbol array (see inset above for an example of the stimulus away as it appears during scanning). Load levels were pseudo-counterbalanced across runs (e.g., L1, L2, L3, L3, L2, L1). All individuals were required to meet a threshold of 90% accuracy before entering the scanner to ensure that they were able to engage in the task.

#### MRI parameters

MRI images were acquired with parameters that have previously been published in fMRI investigations of cognition in TBI [30–32]. First, 3-D high resolution T1-weighted MPRAGE images (9.9 ms/4.6 ms/8° repetition time/echo time/flip angleTR/TE/FA), 240 X 204 X 150 mm^3^ field of view (FOV), 256 X 205 X 150 acquisition matrix, two averages, was acquired to provide high resolution underlays for functional brain activation. Functional imaging data were echo planar imaging (EPI) consisting of: 2,000ms/30 ms/89°, TR/TE/FA, 230 X 230 mm^3^ FOV, 80 X 80 acquisition matrix, thirty-four 4-mm-thick axial slices with no gap between slices.

#### fMRI data preprocessing

The first four volumes of each run were removed from analyses to control for signal instability, resulting in a final time series of 240 volumes per run (i.e. 480 total volumes). All preprocessing steps were conducted using SPM8 (http://www.fil.ion.ucl.ac.uk/spm/software/spm8/). For all volumes, bad slices were first repaired using art-slice procedure using the ArtRepair toolbox [33]. The volumes were then slice time corrected and realigned using SPM8. Spike artifacts were identified and eliminated with despiking filters available in ArtRepair toolbox using a 17-point moving average of the unfiltered data. High pass filtering was bypassed at this level to permit analysis of high to low frequency in the component structure within the Group ICA for fMRI Toolbox (GIFT; http://mialab.mrn.org/software/index.html). Each subject’s high-resolution (1mm x 1mm x 1mm) TI image was co-registered to the mean functional image using SPM8. The co-registered T1 image was then segmented using SPM8, which produced a normalized (MNI space) gray matter image. The functional images were then warped to the MNI space using the parameters computed from the T1 image. The normalized functional volumes and the gray matter image were resliced into 3 mm x 3 mm x 3 mm voxels. To reduce the effect of ringing artifact and to improve signal to noise ratio [34], a 6mm smoothing filter was applied. Strictly as a quality control check for head movement, volume wise assessment of motion was conducted to rule-out individual cases where head motion was greater than 20% of the total volumes (see Table 4). Of note, this volumewise check for motion was for subject removal and volume censoring/interpolation *were not* performed in order to retain the contiguity of the time series and permit identification of variance due to motion within the spatially constrained ICA (see below). ArtRepair detected one TBI subject with significant motion affecting over 20% of the total volumes, and this subject was consequently removed from the study.

**Table 4:** Motion detection summary.

**TBI**	**% of volumes affected**	**Std. Dev. Of Data**	**Current threshold (% of mean)**	**Current threshold (std. dev.)**
**Run 1**	2.39 (3.93)	1.29 (0.39)	6.50 (1.92)	1.30 (0.01)
**Run 2**	2.38 (3.60)	1.31 (0.40)	6.47 (1.86)	1.30 (0.01)
**Healthy Control**	**% of volumes affected**	**Std. Dev. Of Data**	**Current threshold (% of mean)**	**Current threshold (std. dev.)**
**Run 1**	1.09 (3.33)	1.40 (0.41)	5.66 (1.20)	1.30 (0.01)
**Run 2**	0.81 (1.54)	1.34 (0.34)	5.83 (1.26)	1.30 (0.01)

All data represent mean and standard deviation presented as: mean(sd)

#### Volumetric values

To help constrain the interpretation of neural network connectivity, we integrate information about brain volume change over time. White matter (WM), cerebral spinal fluid (CSF), and gray matter (GM) volumes were calculated for each subject using Free Surfer software [35–37]. See Table 5 for a comparison of WM and GM mean and standard deviation values between groups.

**Table 5:** FreeSurfer analysis: Mean (sd) volume ratio.

	TBI	HC
GM/Total	0.40** (0.04)	0.43** (0.03)
WM/Total	0.29* (0.04)	0.31* (0.02)

*Difference between TBI and HC GM significant at p = 0.023

**Difference between TBI and HC WM significant at p = 0.002

### Analytic approach and pipeline

**Fig 2:**
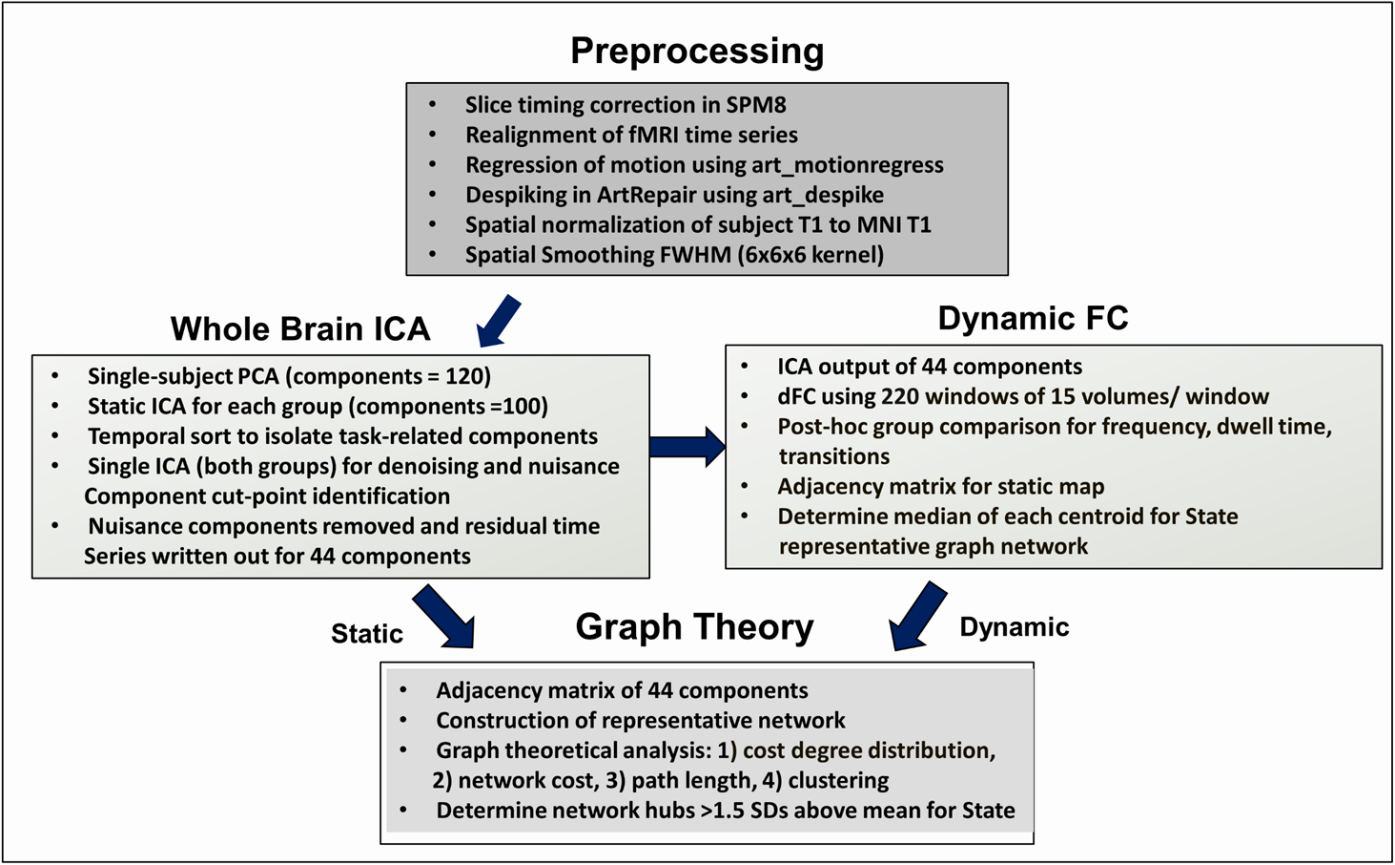
Data processing pipeline for functional data. Abbreviations: ICA: independent components analysis, dFC: dynamic functional connectivity,T1: structural MRI, MNI: montreal neurological institute, FWHM: full width half-maximum.

To test our hypotheses, a functional connectivity network for each subject was developed, and a subject-specific connectivity profile was created based upon an edge-list comparing the correlations of all pairs of viable components outputted by ICA (below). We chose ICA for brain parcellation with the goal of understanding dynamic shifting in large-scale networks and hubs as possible “drivers” of those changes. While the number of nodes may be relatively fewer compared to other methods used in TBI [38,39], this parcellation approach has high reliability by isolating the most robust network nodes and allows large-scale network goals to be addressed [39–41]. The connectivity profile includes metrics fthat summarize the graph output: 1) total functional connections, 2) network strength, 3) network cost, and 4) local and global network efficiency. Fig 2 provides a schematic for the data processing pipeline used in this study.

### Whole-brain mask

We used a whole brain mask from all participants. The segmented gray matter, white matter, and CSF masks were resliced using SPM8 to match the functional files by mapping them onto the average smoothed, normalized functional image for each individual. Resliced gray matter, white matter, and CSF masks were combined for all individuals and the summed image was binarized to create a subject-specific gray matter mask constraining analyses to these voxels [40,42].

### Static and dynamic functional connectivity analysis

Preprocessed data for both groups and both sessions were organized into spatially independent components (ICs) using group-level spatial ICA in GIFT toolbox (http://mialab.mrn.org/software/gift). In the temporal domain, subject-specific data reduction through principal component analysis (PCA) was set to 120 principal components, and consistent with a larger literature including studies in TBI, group data were set to output 100 principal components [4,38,43]. Using ICASSO, the Infomax ICA algorithm was repeated 10 times with minimum and maximum cluster sizes of 8 and 10, respectively.

**Fig 3:**
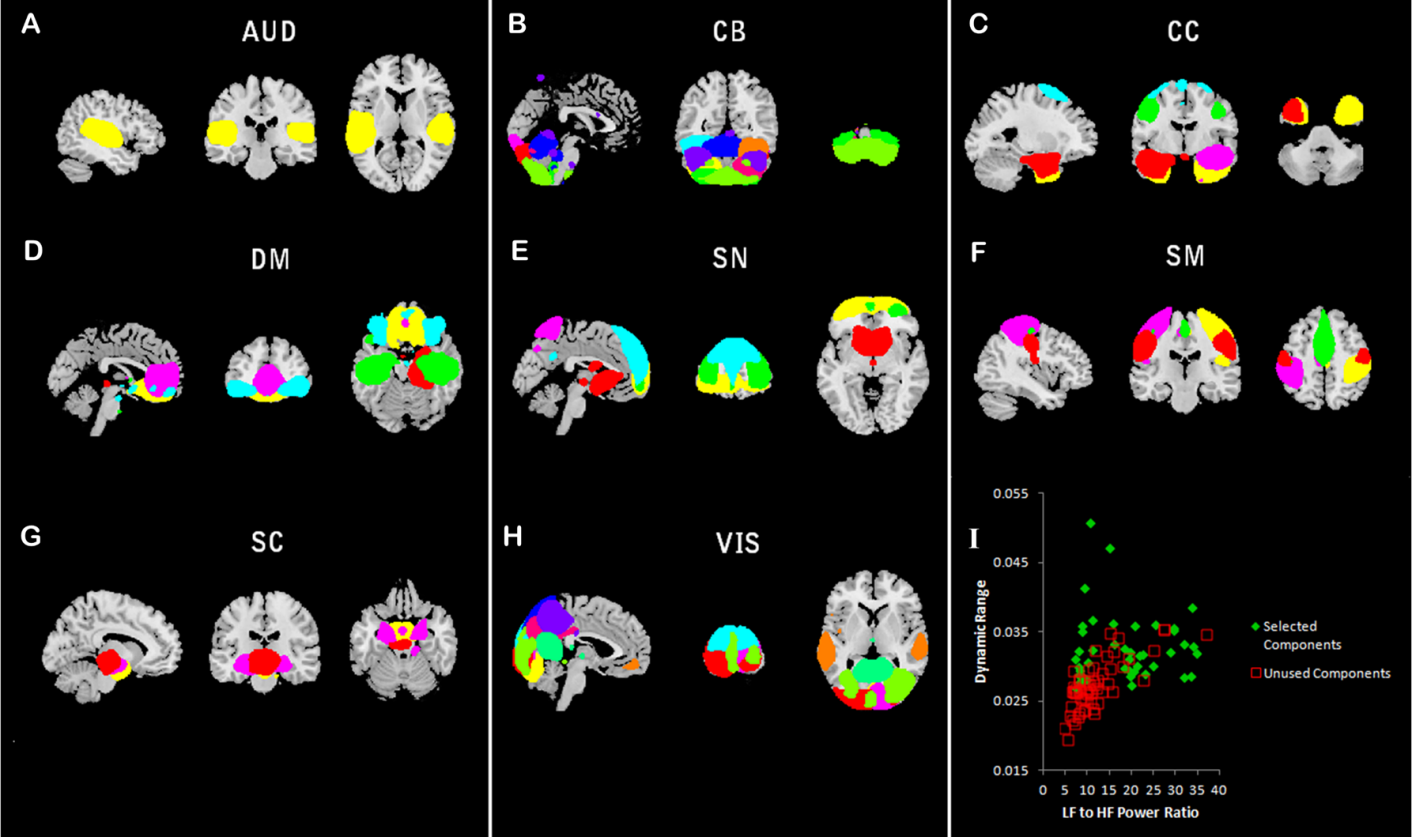
Components from spatially constrained ICA. Illustrates the results of ICA resulting in 44 viable components. Panels A-H reveal the spatial extent of components comprising distinct subnetworks. Panel I shows the dynamic range and low to high frequency ratio used to determine the inclusion of components for the study.

The resulting 100 components were visually inspected and selected based upon four criteria: 1) the ICA time course revealed peak activation in grey matter, 2) the ICA time course showed high ratio of low-frequency to high-frequency fluctuations, 3) the spatial extent of the component maintained a relatively continuous structure (i.e., not fragmented), and 4) had minimal spatial overlap with CSF [41]. In order to permit analysis of a complete network, three components considered “borderline” in quality were included. Functional labels were determined using the Harvard-Oxford atlas and component peak coordinates [44–47] via FSL [40,42]. After comparing all components, three raters reached agreement for the inclusion of 44 components to serve as the primary brain parcellation for both static and dynamic connectivity (see plots for values for dynamic range and high/low frequency ratio for all 100 components in Fig 3, panel I).

### Static network analysis

To provide context for the dynamic analysis, we first conducted a static graph theoretical analysis using the components selected during the ICA output from GIFT (i.e., static network). Graph metrics were determined based upon an FDR-corrected r-value thresholded (p<0.05) connectivity adjacency matrix for the 44 components (i.e., network nodes).

### Dynamic network analysis

Following the initial ICA step which determined the subject-wise static maps, we aimed to assess the dynamic properties of run 1 and run 2 for all subjects. A dynamic functional network connectivity (dFNC) analysis was conducted using temporal dFNC toolbox in GIFT [41]. The 44 spatially independent components for each subject were detrended and low-pass filtered at high frequency cutoff of 0.15Hz. Dynamic functional network connectivity (dFNC) was computed by using a “sliding window” approach which permitted sampling from temporally overlapping windows over the time series. Each session contained 240 volumes per subject, per run, resulting in time courses of 480s (TR = 2s) each. For dynamic approaches, there are decisions to be made regarding the “window size” or the time (or number of volumes) that represent each window of analysis. One important goal in this study was to examine network variation/dynamics after TBI. For task-based studies, a window of ~30 seconds reveals the greatest network flexibility [48], and increasing window sizes reduces variability [49,50]. Therefore, a window step size of 15 volumes (30s) was implemented. This window is also consistent with a literature examining dynamic connectivity using the spatial ICA approach [41] and in at least one study demonstrated that a 30 second time window resulted in the most reliable detection of network communities [51]. Finally, time-varying precision matrices were estimated using LASSO with an L1 regularization to encourage sparsity in connections.

The resulting dFNC windows were clustered and K-mean algorithm based upon Euclidean distance was implemented to cluster the data into six states. As in Allen and colleagues [43], clustering was initially performed using subject exemplars reflecting maximal variability in the data. This step was followed by clustering on the entire time series after initialization with the previous clustering results. The clustering identifies patterns of connectivity or states that are maximally separated from one another. Initially, the number of clusters (i.e. states) were sampled at two to eight clusters. For each trial, cluster occupancy was examined and a setting for six clusters/states was selected based upon the distribution of the state frequencies (e.g., eliminating settings for clusters/states where frequencies for most states were very low, ~2–4%). In the following, we present data for these six independent clusters or states. State-dependent parameters were then output for each subject and each state (e.g., frequency, dwell time, state transitions).

### Neural networks: Metrics from graph analysis

In order to provide context regarding the network topology, several standard graph metrics were analyzed. The following metrics were determined using graph theoretical analysis for both static and dynamic network analysis.

#### Total number of functional connections

The total number of functional connections in the brain for each subject at each time point was evaluated by counting the total number of statistically significant (FDR criterion) functional edges. This metric was used to determine connection density and is used to examine hypotheses regarding edge density and network flexibility/transitions.

#### Network strength

Network strength was defined as the total sum of the absolute value of the network weights. The absolute value was used as a proxy for the magnitude of the synchronization between ROIs. Previous work has shown that a high magnitude of synchronization may be indicative of high metabolic consumption [52]. ROI-strength was defined as the total strength of all functional edges that are incident upon a ROI. We examined these global metrics separately over the two runs to determine their reliability in each group.

#### Local efficiency: Clustering coefficient

In order to examine local communication efficiency within the network, we examine local clustering coefficiency. The local clustering coefficient (CC) of vertex *i* is defined as:

$$C_{i}=\frac{\left|\left\{e_{jk}:v_{j},v_{k}\in N_{i},e_{jk}\in E\right\}\right|}{k_{i}(k_{i}-1)}$$ (1)

where *k*_*i*_ is the total number of neighbors (i.e. neighboring nodes) of node *i*, *N*_*i*_ represents the set of all nodes that are neighbors of node *i*, *E* is the set of all edges in the network, and *e*_*jk*_ represents the connections that exist between the neighbors of node *i*. A high CC indicates that the neighbors of node *i* are well connected, hence suggesting a high local efficiency around node *i*.

#### Global efficiency: Average shortest path length

In order to examine global network efficiency, average shortest path length (PL) was computed between all pairs of nodes. PL is defined as follows:

$$E(G)=\frac{1}{n(n-1)}\sum_{i\neq j\in G}d(i,j)$$ (2)

where n = number of nodes in the network and d(i,j) = shortest distance between nodes *i* and *j*.

If a network is fully connected (hence more efficient), then PL will be 1; otherwise it will be greater than 1. We define global efficiency as the reciprocal of PL.

#### Network cost

Consistent with Roy and colleagues [53], we define the cost of each functional edge as the product of the Euclidean distance between the ROI-pair it connects and the absolute weight of the connection. The overall network cost is determined as the total cost for all edges within the network. Therefore, both network and nodal cost are calculated considering three components: 1) level of synchronization for any edge, or r-value, 2) the number of functional edges, and 3) the physical distance of edges. This allows us to examine whole-brain networks for cost as well as to isolate the most costly brain subsystems when comparing groups.

## Results

### Static connectivity analysis

Fig 3 illustrates the components included during the static and dynamic network analysis. Primary graph metrics included PL and local and global clustering coefficiency (see **Table 6**).

**Table 6:** Global graph metrics for static analysis.

	Mean shortest PL (mean±sd)	Mean local CC (mean ±sd)
**TBI Run 1**	1.319 ± 0.072*	0.718 ±0.07*
**TBI Run 2**	1.315 ±0.069	0.719 ±0.069
**HC Run 1**	1.281 ±0.058*	0.753±0.0579*
**HC Run 2**	1.278 ±0.045	0.741 ±0.044

**Note**: no differences statistically significant at p<0.05.

For context

* = values presented for (p < 0.1) compared to HCs

When examined using Pearson’s correlation, all graph metrics in Table 6 below were highly reliable between runs in both TBI (mean PL: r = 0.86, mean CC: r = 0.86) and HC (mean PL: r = 0.71, mean CC: r = 0.76) samples.

### Dynamic connectivity analysis

Dynamic analysis based upon six network states revealed that states 3, 5, and 6 composed a majority of the total state frequency over the time series (>90% frequency in these three states, see Table 7), whereas states 1, 2, and 4 occurred only in 4 of 23 cases of TBI participants and no HC participants.

**Table 7:** Dynamic connectivity network states.

	State 1 Frequency mean ±sd	State 2 Frequency mean ±sd	State 3 Frequency mean ±sd	State 4 Frequency mean ±sd	State 5 Frequency mean ±sd	State 6 Frequency mean ±sd
**TBI Run 1**	0.043 ±0.20	0.047 ±0.20	0.464 ±0.35	0.043 ±0.20	0.275 ±0.27	0.127 ±0.21*
**TBI Run 2**	0.043 ±0.20	0.087 ±	0.503 ±0.36	0.043 ±0.20	0.19 ±0.23*	0.134 ±0.23
**HC Run 1**	0.0 ±0	0.0 ±0	0.344 ±0.326	0.0 ±0	0.398 ±0.32	0.257 ±0.25*
**HC Run 2**	0.0 ±0	0.0 ±0	0.409 ±0.34	0.0 ±0	0.364 ±0.30*	0.227 ±0.22

**Note**: no between-group comparisons for states 1, 2, and 4; for state 1: n = 1; state 2: n = 3, state 4: n = 1; Abbreviations: sd = standard deviation

*p<0.05.

#### Reliability of dynamic analysis

**Fig 4:**
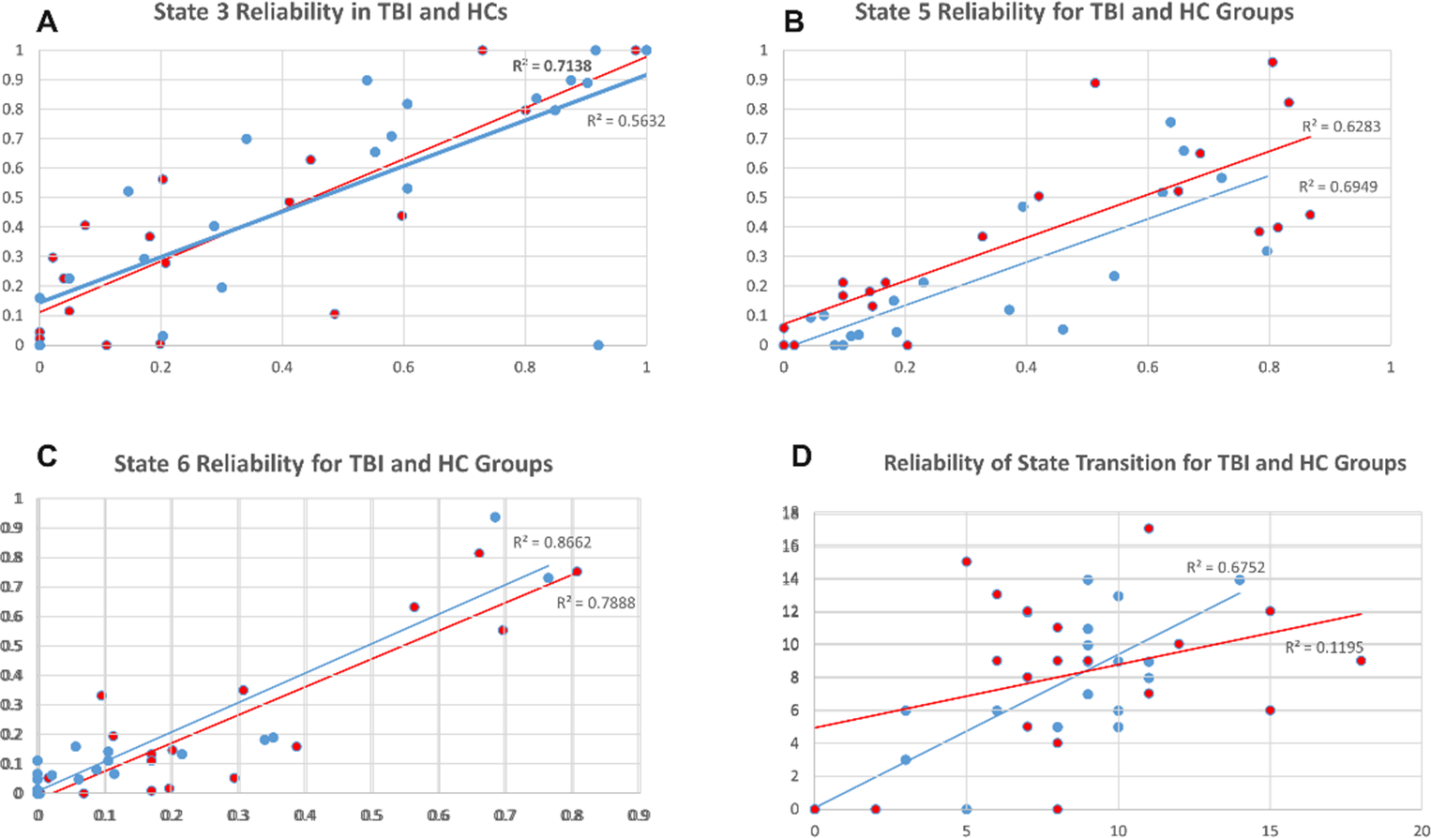
Test-retest reliability across runs for state frequency and transitions for both samples. **Panels A-C**: show the test-retest reliability for the frequency data for states 3, 5, and 6. For both samples, the frequency for a state during run 1 was highly predictive of the frequency for that state in run 2. **Panel D:** provides test-retest reliability for the number of transitions between run 1 and run 2. Transitions in run1 predict transitions in run 2 only for the TBI sample.

**Fig 5:**
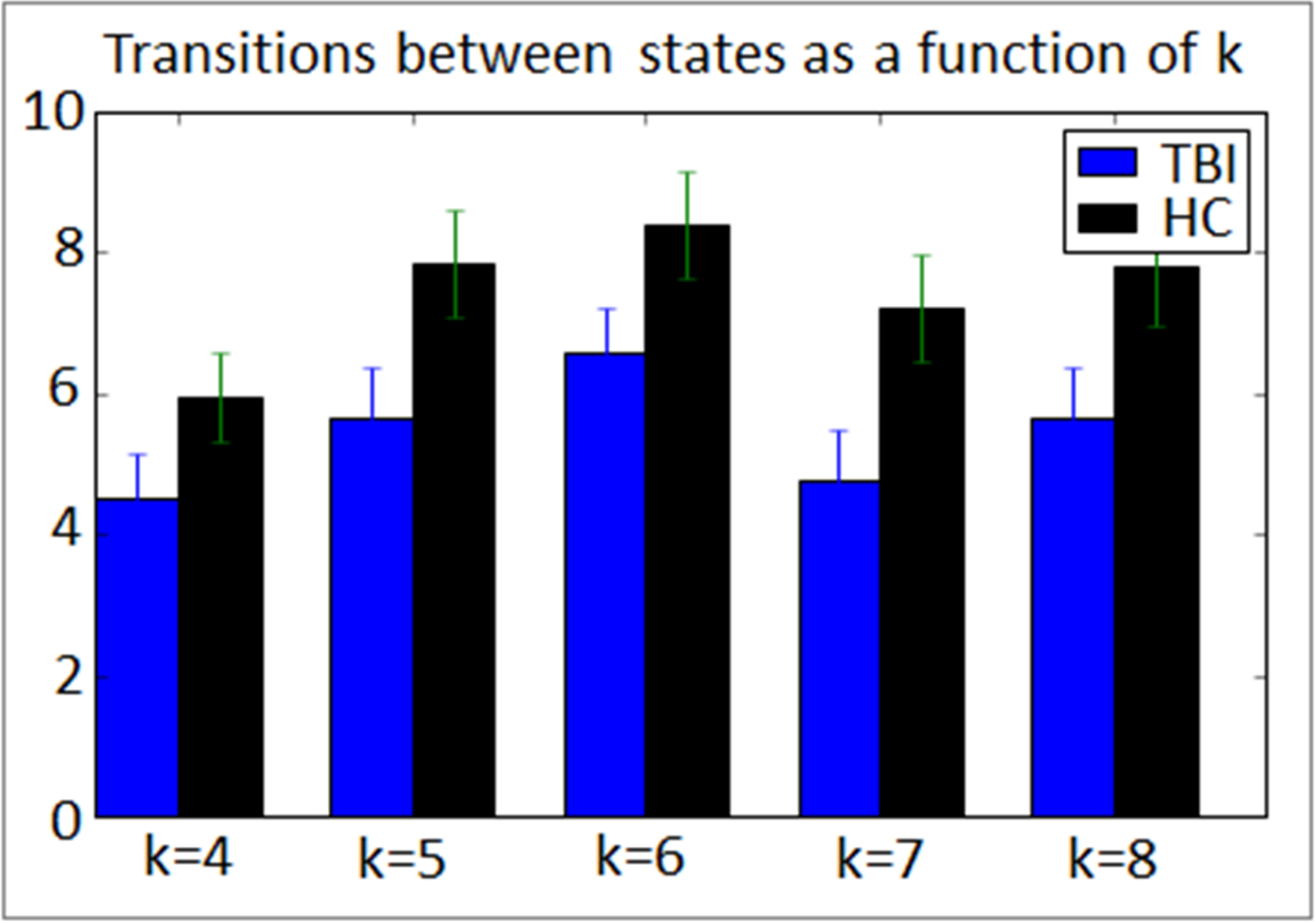
Examining the influence of the number of states (*k*) on state transitions between groups. The TBI sample reliably shows reduced transitions irrespective of investigator decisions regarding *k*. Error bars indicate standard error. * = between group difference significant, p<0.1, ** = between group difference significant, p<0.05.

To examine the reliability of dynamic meta-states after injury (Hypothesis 1), we correlated the frequency and number of state transitions between Run 1 and Run 2 in each sample. Fig 4A–4C reveal that at the individual subject level the frequency for entering a given state during Run1 was highly predictive of the frequency for that state in Run 2 (r-values ranging from 0.785–0.86). However, the number of network transitions was highly comparable between runs for the TBI sample only (r = 0.82, p<0.05); the number of state transitions during Run 1 was a much weaker predictor of the number of transitions for Run 2 in the HC sample (r = 0.34; Fig 4D). Regarding the reliability in state frequency, these data are important and confirm within-subject reliability in observable brain states over time. However, the lack of reliability in transition data in the HC sample are interesting and possibly point to less dynamic range in the TBI sample compared to the HC sample. Given that the number of possible states (*k*) is investigator determined, we examined directly the influence of *k* on state transitions. Fig 5 reveals consistent results for fewer state transitions in TBI irrespective of the number of states permitted at the first step. These findings regarding reduced state transitions are discussed in detail below.

#### Comparing dynamic states between groups

**Fig 6:**
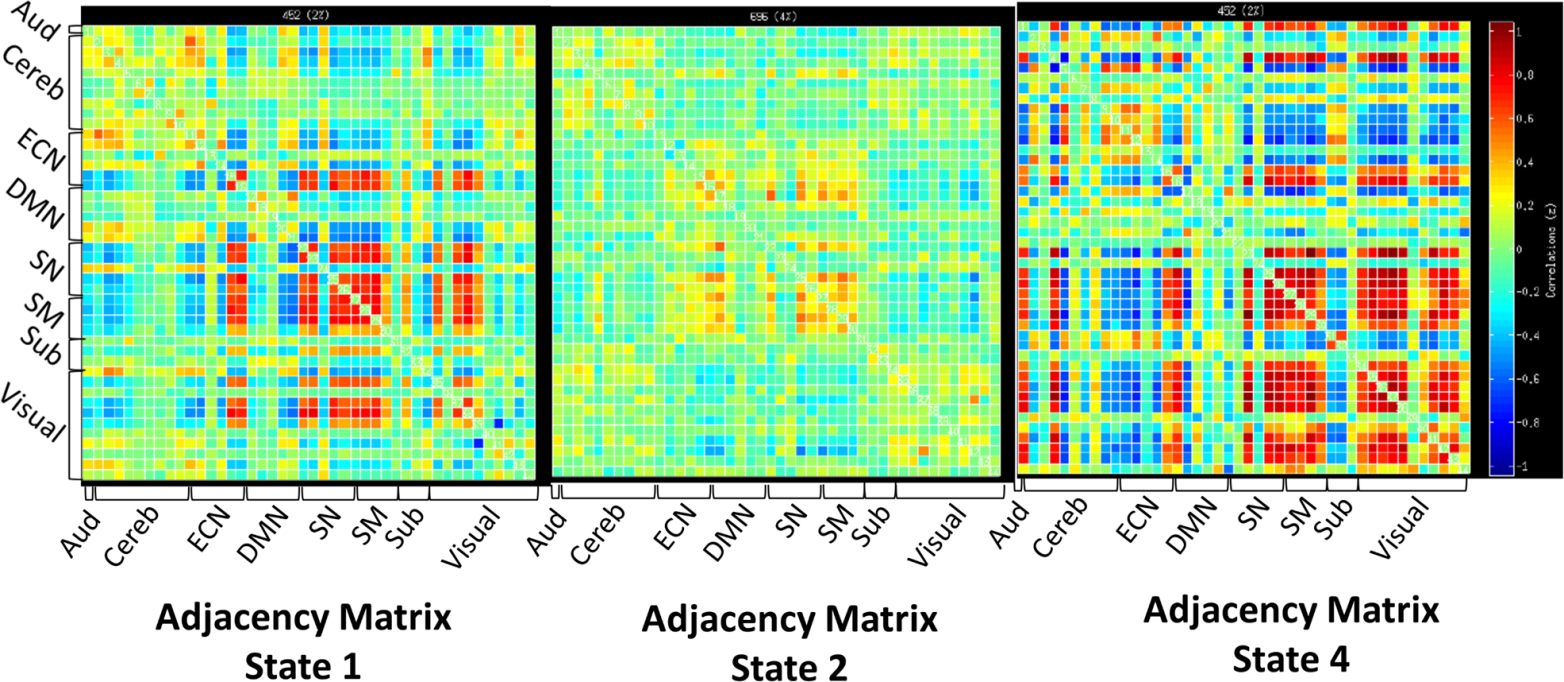
Adjacency matrices for network states 1, 2, and 4. Adjacency matrices for states 1, 2, and 4 which appeared only in the TBI sample. Abbreviations: Aud: auditory network, ECN: executive control network, DMN: default mode network, SM: sensory-motor network, SN: salience network.

**Fig 7:**
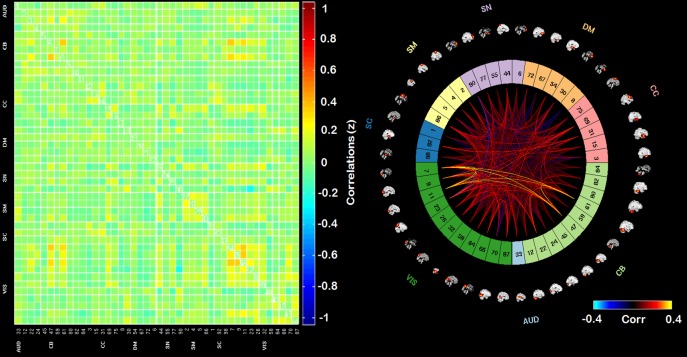
Adjacency matrix and connectogram highlighting the connectivities across subnetworks for state 3. Abbreviations: Aud: auditory network, ECN: executive control network, DMN: default mode network, SM: sensory-motor network, SN: salience network.

**Fig 8:**
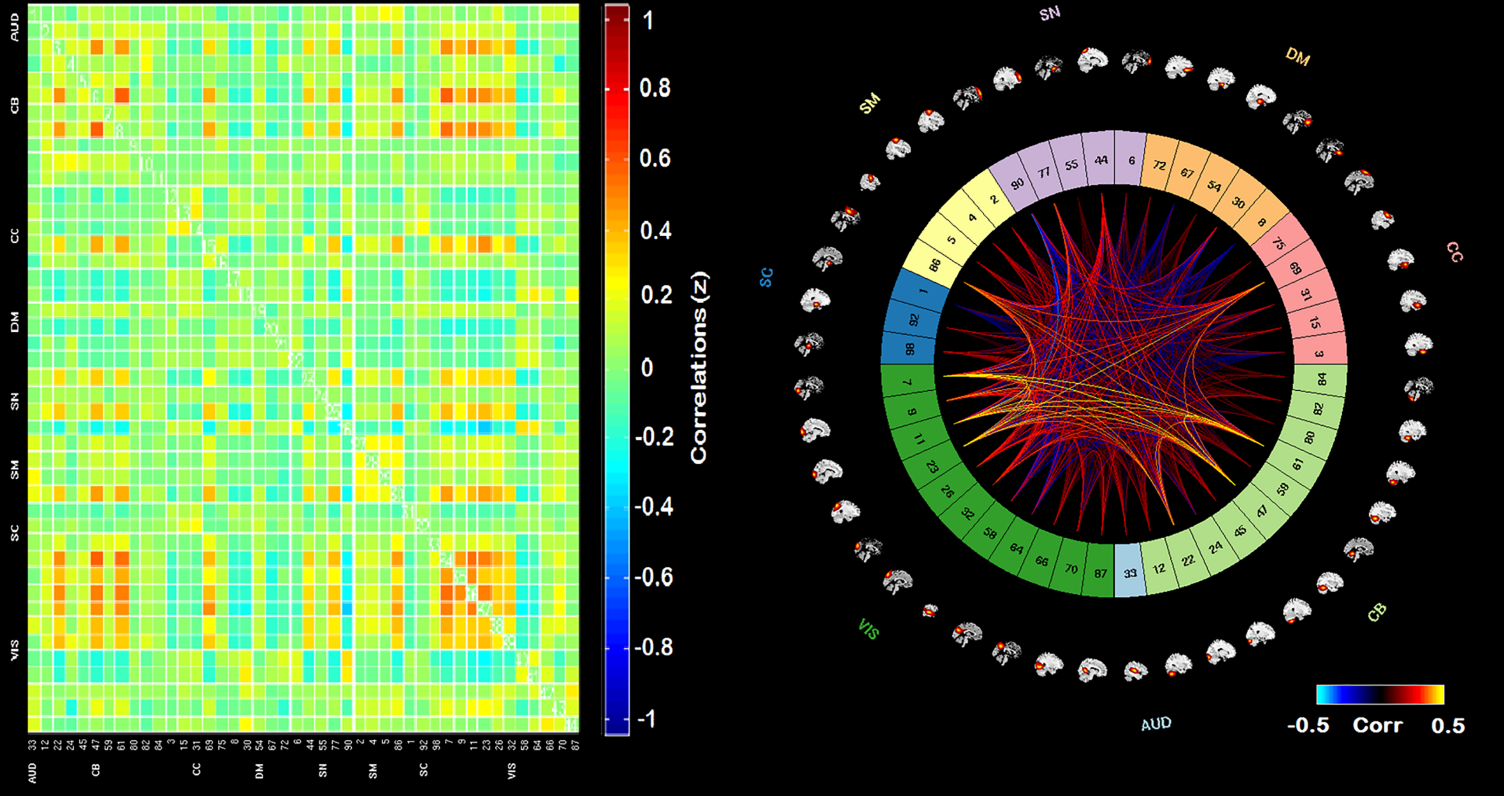
Adjacency matrix and connectogram highlighting the connectivities across subnetworks for state 5. Abbreviations: Aud: auditory network, ECN: executive control network, DMN: default mode network, SM: sensory-motor network, SN: salience network.

**Fig 9:**
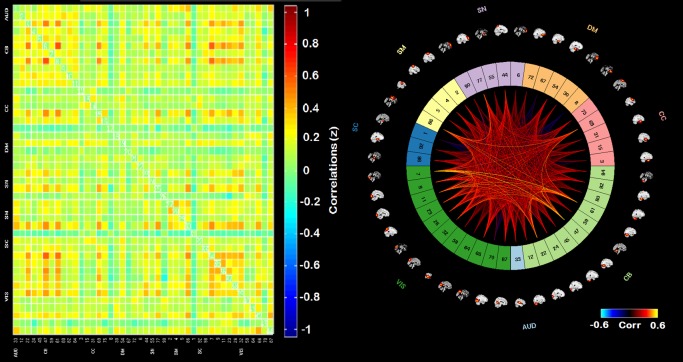
Adjacency matrix and connectogram highlighting the connectivities across subnetworks for state 6. Abbreviations: Aud: auditory network, ECN: executive control network, DMN: default mode network, SM: sensory-motor network, SN: salience network.

The adjacency matrices for the least visited states (1, 2, and 4) are presented in Fig 6. Figs 7–9 present the adjacency matrices and connectogram for the most highly visited states (states 3, 5, and 6). States 5 and 6 showed the highest state frequency in the HC sample (13/19 participants) compared to the TBI sample where state 3 maintained the highest frequency and states 5 or 6 were less commonly dominant (6/20 participants) [χ^2^(2), n = 39) = 5.75, p < .05].

Given the finding that states 5 and 6 differentiated the groups, we correlated mean frequency for these two states with three behavioral outcomes variables in TBI: 1) mean RT during the in-scanner task (mDSST), and performance on two tasks emphasizing speeded information processing outside the scanner (VSAT and Trails A). The results revealed stronger relationships between state 5 frequency in the TBI sample, (mDSST RT: r = -0.25, p = 0.27; VSAT: r = 0.524, p = 0.021; Trails A: r = -0.379, p = 0.12) and non-significant findings for state 6 (mDSST RT: r = -0.12, p = 0.582; VSAT: r = 0.111, p = 0.651; Trails A: r = -0.290, p = 0.243), where the results for all 6 comparisons revealed better performance with increased state frequency.

### Network hubs as drivers of network states and performance

**Fig 10:**
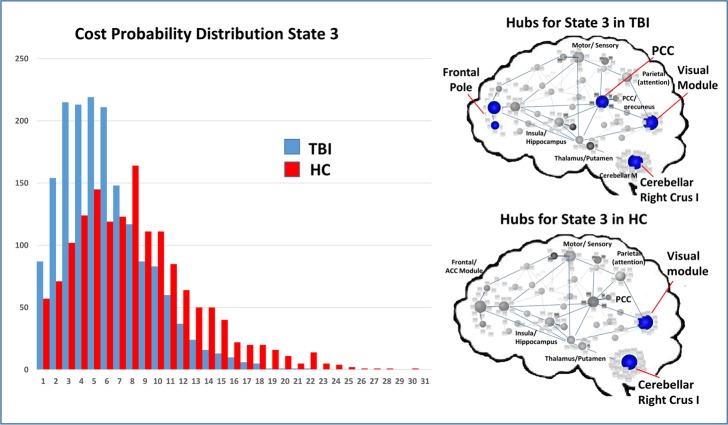
Cost probability distributions and hubs for state 3 for both samples. **Left**: Cost probability distributions for state 3. Distributions based upon the cost for all nodes collapsed across runs for each group. **Right**: Hubs identified for each of the samples for state 3.

**Fig 11:**
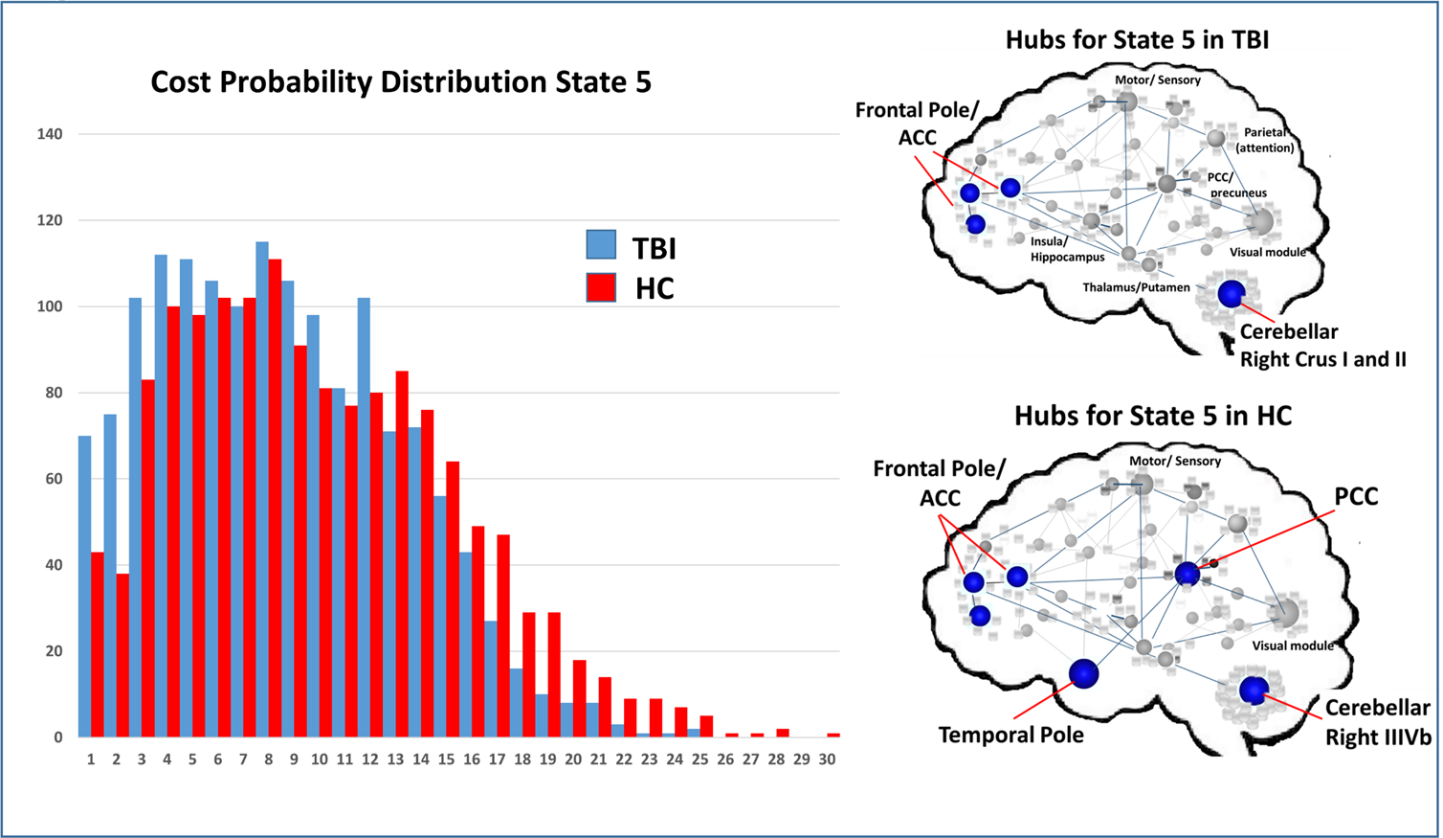
Cost probability distributions and hubs for state 3 for both samples. **Left**: Cost probability distributions for state 5. Distributions based upon the cost for all nodes collapsed across runs for each group. **Right**: Hubs identified for each of the samples for state 5.

**Fig 12:**
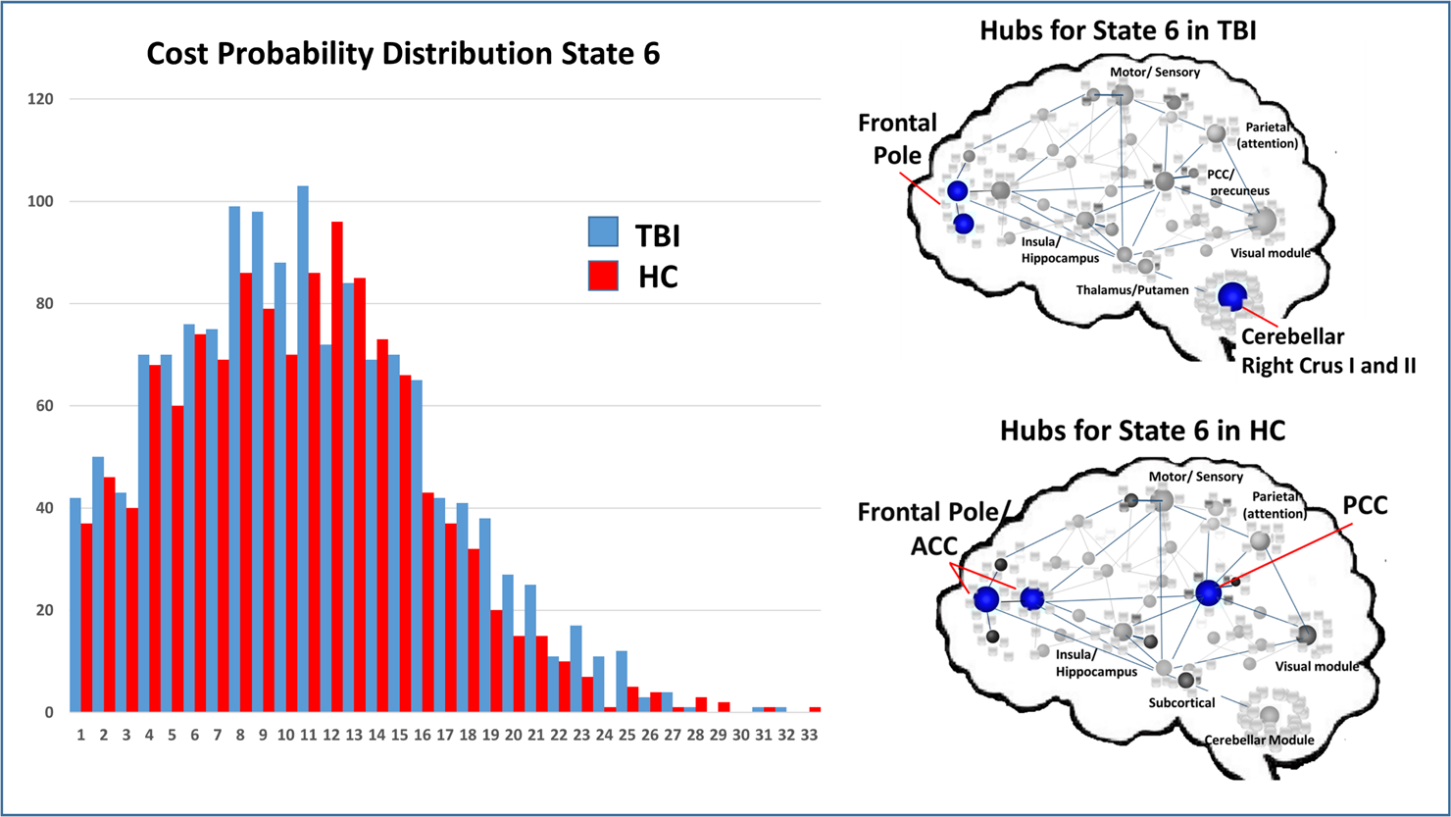
Cost probability distributions and hubs for state 3 for both samples. **Left**: Cost probability distributions for state 6. Distributions based upon the cost for all notes collapsed across runs for each group. **Right**: Hubs identified for each of the samples for state 6.

To test Hypothesis two, we aimed to determine if global measures of connectivity or local hubs within subnetworks had influence on the frequency of network states, operating to “drive” network frequency. To isolate the top 4–5 most highly connected nodes for each state, we used a simple measure identifying highly connected hubs (>1.5 SDs above the mean for each state in each sample); Figs 10–12 illustrate the cost distributions and most highly connected regions for network states 3, 5, and 6. Given the high frequency of state 5 in HCs and that this state was an important differentiator between groups, we examined the relationship between the mean cost for state 5 hubs (see Fig 11) and the frequency for state 5. That is, as part of the second hypothesis, we aimed to determine if state-level dynamics could be driven by the connectivity of the most highly connected nodes. Only partial support for this hypothesis was revealed. Of the three hubs of interest, nodes in the SN showed some relationship with state 5 frequency, whereas nodes in the DMN and ECN were not strong predictors or drivers of state 5 frequency (Table 8). This indicates that the hub regions chosen for analysis may only selectively serve as “drivers” of brain states movement between network states.

**Table 8:** Correlation between state 5 hubs and state 5 frequency in TBI.

	Frequency State 5 run 1	Frequency State 5 run 2	Frequency State 5 avg.
**State 5 Hubs (cost) run 1**	r = 0.506, p = 0.032*	r = 0.593, p = 0.009*	r = 0.579, p = 0.012*
**State 5 Hubs (cost) run 2**	r = -0.009, p = 0.974	r = 0.238, p = 0.376	r = 0.119, p = 0.661
**State 5 Hubs (cost) avg**	r = 0.380, p = 0.147	r = 0.259, p = 0.332	r = 0.457, p = 0.075

Examining the primary hubs in state 5 (collapsed and averaged) to determine if they are “drivers” of state 5 frequency in TBI. Average cost for hubs for run 1 and run 2 and then the average between runs. Average cost taken for 5 most highly connected nodes for state 5 in TBI (2 in SN, 2 in cerebellum, and 1 in DMN).

*p<0.05.

To determine if the network cost associated with state 5 hubs predicted behavior, correlational analysis was conducted using the three simple visual scanning tasks (mDSST, Trails A, VSAT) and hubs established state 5. These three hubs showed inconsistent relationships with comparisons revealing small to medium effect sizes, with the hubs of the SN and ECN showing some prediction of behavior and no relationship between DMN and behavior. Overall, network hubs in the DMN, SN, and ECN were generally poor to modest predictors of performance on tests of information processing efficiency collected inside and outside the scanner (see Table 9).

**Table 9:** Correlation between state 5 hubs cognition in TBI.

	mDSST reaction time (avg)	VSAT total score	Trails A (time)
**Avg Cost Anterior DMN**	r = 0.100, p = 0.733	r = 0.051, p = 0.862	r = -0.10, p = 0.973
**Avg Cost: Anterior SN (ACC/insula)**	r = 0.183, p = 0.531	r = 0.43, p = 0.128	r = -0.375, p = 0.207
**Avg Cost: ECN (Temporal Pole)**	r = -0.415, p = 0.110	r = 0.086, p = 0.760	r = -0.559, p = 0.038*

Examining the relationship between cost in three hubs in a priori networks and behavioral variance on the in-scanner task (mDSST) in the TBI sample (Note: Nodes from DMN and SN were >1.5 sd above the mean; ECN node was 1sd above the mean). Abbreviations: VSAT = visual search and attention test, mDSST = modified digit symbol substitution task, RT = reaction time, ECN = executive control network, DMN = default mode network, ACC = anterior cingulate cortex.

* = p < .05.

### Examining network dynamics (transitions)

One important goal in this study was to determine the predictors of network variability, or state transitions, and the relationship between network variability and performance. Subtle differences were evident when comparing the number of state transitions between the TBI and HC groups. The mean number of transitions in the TBI sample was generally lower for both runs (Run 1 mean = 6.7, sd = 4.1; Run 2 mean = 6.4, sd = 4.8) compared to the HC sample (Run 1 mean = 8.5, sd = 4.2; Run 2 mean = 8.2, sd = 4.8; p = 0.06). To examine the consistency of these results we directly examined the influence of k on state transitions and the TBI sample consistently showed reduced network dynamics (see Fig 5). We interpret these data to be indicative of reliably reduced state transitions in TBI. This finding does not support the hypothesis that brain injury results in increased network variability. Given the variability in age and time post injury range within the TBI sample, we examined the influence of these factors on state transitions using bivariate Pearson’s correlations; neither were strong predictors of the number of transitions between states (age: r = 0.28; time post injury: r = 0.17).

In order to assess the relationship between network dynamics and performance variance (testing Hypothesis 3a), we correlated the standard deviation of the in-scanner performance (performance variance influence) with the number of transitions between states (network dynamics/variance) for both runs 1 and 2. The findings are presented in Table 10 (top), but overall, the number of run 1 transitions was a modest predictor of network dynamics for variance in performance for both behavioral runs. That is, early network transitions predicted variance in performance for run 1 and run 2.

**Table 10:** Examining network dynamics (transitions) after TBI.

	State Transitions Run 1	State Transitions Run 2
**mDSST run 1 (sd)**	r = -0.440, p = 0.046**	r = -0.155, p = 0.502
**mDSST run 2 (sd)**	r = -0.416, p = 0.060*	r = -0.215, p = 0.350
**Mean Nodal Degree Run 1**	r = -0.457, p = 0.028**	r = -0.265, p = 0.222
**Mean Nodal Degree Run 2**	r = -0.525, p = 0.010**	r = -0.352, p = 0.099*

**(Top):** The relationship between the number of state transitions and variability on in-scanner task performance. **(Bottom):** The relationship between state transitions and mean nodal degree (total network connectivity). Transitions during the first run is a predictor of variance for both runs, whereas state transitions during the second run did not predict behavioral variance.

*p<0.10

**p<0.05.

Lastly, it was a goal to determine if local or global connectivity predicted the number of transitions. Based upon the static network cost we examined the mean connectivity of the top five nodes (i.e., local hubs, >1.5 sd) to represent regional influence on state transition and mean nodal connectivity across the network as a measure of global influence on state transition. Regional influences maintained no relationship with state transitions (r-values ranging from 0.07–0.10), but global connectivity during run 1, measured as the mean nodal connectivity for all nodes, was an important predictor of state transitions for both runs 1 and 2 (see Table 10 bottom).

## Discussion

The current study used a graph theoretical framework and dynamic connectivity modeling to examine functioning of large-scale neural networks after head trauma. We used two runs of task-related data collection in order to examine the reliability of the findings. With regard to reliability, it should be emphasized that at the individual subject level, the frequency for entering and dwelling within distinct brain states identified using this dFC approach was highly reliable over the course to two extended fMRI data acquisition periods (each ~8 minutes). The following discussion is focused on several key points. First, analysis of network hubs revealed that they only inconsistently served as “drivers” predicting brain states. Second when examining distinct networks, there were network states unique to the TBI sample; roughly 1/6^th^ of the sample showed evidence of states not evident in the HC sample. Third, it appears that the TBI sample may be less likely to transition between states and the number of transitions during run1 was a modest predictor of performance variability for both runs. Finally, the network variance (identified as state transitions) was predicted by global connectivity, indicating that the increased connectivity commonly observed in TBI may be a source of reduced network variability. We discuss the implications these findings have for understanding large-scale neural network changes occurring after significant neurological disruption.

### Hubs as network drivers

There is a growing literature demonstrating the unique role of network hubs as driving brain dynamics toward specific states [15,23,54,55]. In Hypothesis one, we anticipated that network hubs would predict the frequency for dynamic connectivity states. We focused the analysis on state 5 given its relatively high frequency and that it was one state that differentiated the groups. There was mixed support for this hypothesis for a network “hub” based upon the number of connections as opposed to the location within the network (i.e., average degree >1.5 standard deviation of the sample). Five hubs were examined and collapsed into three distinct networks: the DMN, the SN, and the cerebellum. These findings revealed that, within individuals with TBI, mean degree for state 5 hubs maintained a positive correlation with state 5 frequency for run 1 but not for run 2. Given our goal to use the two runs of data to demonstrate the reliability of findings, interpreting this finding is difficult. When examining the reliability of each of these metrics (state 5 frequency and state 5 hub cost) we find the reliability of *frequency* for state 5 between runs in the TBI sample was very high (r = 0.83), but there is greater variability in the reliability of *cost* for state 5 hubs between runs (r = 0.57). If we focus on possible effects of chronology, hubs may serve as the backbone for information transfer during run 1, thus driving activity in state 5, but the network requirement for this influence diminishes over time. Similar network dynamics have been observed in schizophrenia where early measures of network flexibility during early runs predict concurrent and later network functioning including behavioral performance [56]. Overall, the hypothesis that network hubs act as “drivers” of brain states was partially supported and the data here may indicate that early hub connectedness may be predictors of brain states for more protracted windows of time.

While our goal in this study was to focus on cortical hubs as drivers of network states post injury, one unexpected finding worth revisiting was the repeated observation that crus 1 and II functioned as a hub within the network. This was evident for all three states showing the highest frequency post TBI and in state 6, this finding was unique to the TBI sample. The cerebellum is involved in a number of functions including timing and circadian rhythms, associative learning mechanisms, and higher level cognitive processing (see [21,57] for meta-analytical and theoretical reviews). In particular, Crus I and II have been consistently linked to roles in information processing (e.g., working memory), so the finding that these were sites as hubs in the current task-based network analysis is intriguing. We anticipate that further investigation here is worthwhile given that the history of findings of enhanced cerebellar response in recent network connectivity modeling in both moderate and severe TBI [58] and mild TBI [17,59] and even altered cerebellar response as a primary indicator of response to methylphenidate intervention to improve cognition in TBI [32,60].

### State transitions and behavioral variability

The primary prediction that brain injury results in greater variability (i.e., transitions) in brain states was partially supported. Some support was observed in cases where the TBI sample entered brain states not evident in the HC sample, which is further discussed below (High Cost Network States). However, for both runs, the TBI sample showed reduced network dynamics (fewer state transitions) and maintained lower frequency for states common in HC (states 5 and 6), instead showing a mean frequency for a single state at 48% (state 3), higher than any state frequency in the HC sample. Therefore, the range of network expression was more restricted in the TBI sample with respect to: 1) the number of transitions between states and 2) the frequency states outside of state 3, including the most common state in HCs (state 5). Given the established literature documenting the increased variation in behavioral performance during tasks of cognition [61] and motor functioning [6] after injury, we expected that these behavioral deficits would be mirrored by more variable network dynamics (operationalized here as transitions). In contrast, however, post-hoc analysis of these effects revealed that mean standard deviation in RT while performing the task in the scanner during run 1 was a negative predictor of the mean number of state transitions across both runs (r-value = -0.440 to -0.416; Table 10 top). In other words, as the variability in performance increases, individuals with TBI were *less likely* to show state transitions. Other investigators have reported similar results after injury demonstrating that after TBI, higher brain signal variability may be predictive of cognitive recovery [62] and that brain injury results in reduced network variability [63]. Recent work based in control theory used simulations demonstrate a limited dynamic range of states available to individuals with mild TBI [64]. This finding could be related to a more established literature demonstrating increased use of resources or “diffusion” of the BOLD response during task activation studies (for review see [30,65]). The loss of nodal specificity may result in incorporation of additional resources or engagement of alternative auxiliary pathways [24,66] or hubs (see Fig 10 in TBI) resulting in less freedom for expression of network dynamics and greater susceptibility to neural noise [64]. This “hyperconnectivity” response observed in other samples [5,14,53,67] may operate to accommodate ongoing task demands in the context of reduced neural resources, but one consequence may be reduced network flexibility.

Finally, while there were subtle relationships between the states differentiating the two groups (states 5 and 6), the relationship between network dynamics and cognitive outcome does not appear to be straight-forward. Future work should be organized around modulating variation in performance using task-load manipulations to determine the contributors to network states arising during task that may account for performance variability. Similar analyses might be extended to other clinical disorders, such as multiple sclerosis, where the diffuse effects of pathophysiology have been shown to result in increased performance variability.

### High cost network states in TBI

One final observation is that four of the 23 subjects with TBI showed zero frequency of time spent in states 5 and 6 and very little to no frequency in state 3. These subjects occupied states 1, 2, and 4 and three of the four showed zero transitions between states. In each case, occupation of these rare brain states was true for both runs so the finding was reliable, but given the small numbers observed in these states, it is possible that these three rare states were occupied solely TBI cases as opposed to HC cases only by chance. If allowed to interpret the networks for these rare cases, states 1 and 4 were characterized by high overall network cost with several regions showing very high connectivity including the ECN, SN, and visual networks (See Fig 6). Comparing this sub-group to the remainder of the TBI sample did not reveal any striking differences with regard to age or education (ages 20–34, 3 with 12-years education, 1 with 18 years education), though reaction time during the fMRI task and during the VSAT was modestly slower (~100 ms). The reason for the emergence of these states in this TBI sub-group is not clear, but based upon clinical MRI at the time of injury, there was significant disruption of frontal systems in these four cases. Additional work will be needed to determine if state-level analyses reveal sub-types within TBI that emerge as distinct network responses to injury or if these states are not related to pathology and are also visible in HCs.

### Study conclusions and limitations

The current approach provides an important opportunity to examine whole-brain connectivity over multiple time scales. While we report the first set of findings related to the local and global changes in network connectivity and cost in moderate and severe TBI, this study is not without limitations. First, like most all studies in this literature, TBI is a heterogeneous disorder and ideally we would have a sample size that permitted subgroups for analysis. While the current data demonstrated the within subject reliability of network dynamics and the sample size here is comparable to prior graph theory analysis examining static networks after moderate and severe TBI [4,6,7,68], the sample size for this study does preclude direct examination of the reliability of these findings with respect to the groups (e.g., split-half reliability). For this reason, replication of the current findings is needed in a separate group of individuals with moderate and severe TBI with focus on the primary findings: 1) network dynamic loss (i.e., reduced state transitions), 2) occurrence of rare states not evident in the healthy adults in this sample. Second, ICA has distinct advantages both with respect to reduction of the influence of nuisance signal (17) and avoiding signal averaging across heterogeneous signals. However, there are inherent limitations to a brain parcellation of 44 network nodes in particular with respect to subtle effects within subnetworks (e.g., distinct functional roles of the PCC). To the degree that BOLD fMRI is sensitive to these subtle differences, group-level ICA may not detect more nuanced effects where the network nodes are changing as well as the between-node interactions. However, we anticipate that ICA remains an ideal statistical application given the focus in this study on large-scale network dynamics, including observing the most reliable networks (e.g., DMN, SN). In summary, this study supports the reliability of examining dynamic network states after neurological disruption. It also supports prior work demonstrating the potentially unique role of some network hubs in motivating network states. Finally, the possible loss of network dynamics after TBI may be a predictor of greater behavioral variability. This link between network dynamics and behavioral outcome in TBI appears to be an important future line of investigation.
